# Hormonal contraceptives impair psychomotor vigilance task performance under sleep deprivation with stress induction

**DOI:** 10.1093/sleepadvances/zpag052

**Published:** 2026-05-08

**Authors:** Kathryn E R Kennedy, Alisa Huskey, David C Negelspach, Jungwon Cha, Salma I Patel, Brieann C Satterfield, William D S Killgore

**Affiliations:** SCAN Lab, Department of Psychiatry, University of Arizona College of Medicine – Tucson, University of Arizona, Tucson, AZ, United States; SCAN Lab, Department of Psychiatry, University of Arizona College of Medicine – Tucson, University of Arizona, Tucson, AZ, United States; SCAN Lab, Department of Psychiatry, University of Arizona College of Medicine – Tucson, University of Arizona, Tucson, AZ, United States; SCAN Lab, Department of Psychiatry, University of Arizona College of Medicine – Tucson, University of Arizona, Tucson, AZ, United States; Mel and Enid Zickerman College of Public Health, University of Arizona, Tucson, AZ, United States; University of Arizona College of Medicine – Tucson, University of Arizona, Tucson, AZ, United States; Sleep and Performance Research Center, Washington State University, Spokane, WA, United States; Department of Translational Medicine and Physiology, Elson S Floyd College of Medicine, Washington State University, Spokane, WA, United States; SCAN Lab, Department of Psychiatry, University of Arizona College of Medicine – Tucson, University of Arizona, Tucson, AZ, United States

**Keywords:** women’s health, hormonal contraceptives, sleep deprivation, hormones, sex differences, cognition, stress, psychomotor vigilance

## Abstract

**Study Objectives:**

Hormonal contraceptives (HC) have been shown to influence cognitive performance, yet their effects under conditions of sleep deprivation remain poorly understood. Consistent with prior research demonstrating greater fatigue among individuals using HC, we hypothesized that vigilant attention would be impaired among females using HC compared with males and females not using HC.

**Methods:**

Forty-eight participants (18–30 years; 23 females) underwent 28 hours of total sleep deprivation (TSD) while completing serial stress tasks, psychomotor vigilance tasks (PVT), and providing salivary cortisol samples. Mixed-effects models were constructed with either PVT outcomes or cortisol reactivity as dependent variables, group (males, females not using HC, females using HC) time, and mood induction group as fixed effects, as well as a group-by-time interaction, and participant as a random effect.

**Results:**

Females using HC demonstrated significantly more PVT lapses (F(1,45) = 3.75; *p* = .031) and a trend toward lower PVT speed (F(2,44) = 3.05; *p* = .057) than males and females not using HC during the second half of the sleep deprivation period. No significant group differences were observed for PVT reaction time or false starts. Neither self-reported sleepiness, cortisol reactivity, or amplitude differed by group.

**Conclusions:**

Use of HC was associated with greater attentional instability following TSD, reflected by increased lapses and reduced vigilance efficiency. These effects occurred independently of cortisol responses. Larger studies are needed to further characterize the cognitive effects of HC use under sleep loss and to elucidate underlying mechanisms.

**Statement of Significance**

Steroid sex hormones, such as estrogen and progesterone, exhibit 24-hour rhythms and fluctuate throughout the menstrual cycle in females of reproductive age. These rhythms are altered among individuals using hormonal contraceptives and relatively little is understood about how this influences cognition. Even less is understood about how these medications influence sleep or cognitive performance under sleep deprivation. In this study, individuals using hormonal contraceptives demonstrated significantly poorer performance on a psychomotor vigilance task than males or females not using hormonal contraceptives during the latter half of a 28-hour total sleep deprivation period. These findings highlight the need for further investigation into the effects of hormonal contraceptives on cognitive functioning, particularly under conditions of sleep loss.

## Graphical Abstract

**Graphical Abstract:**
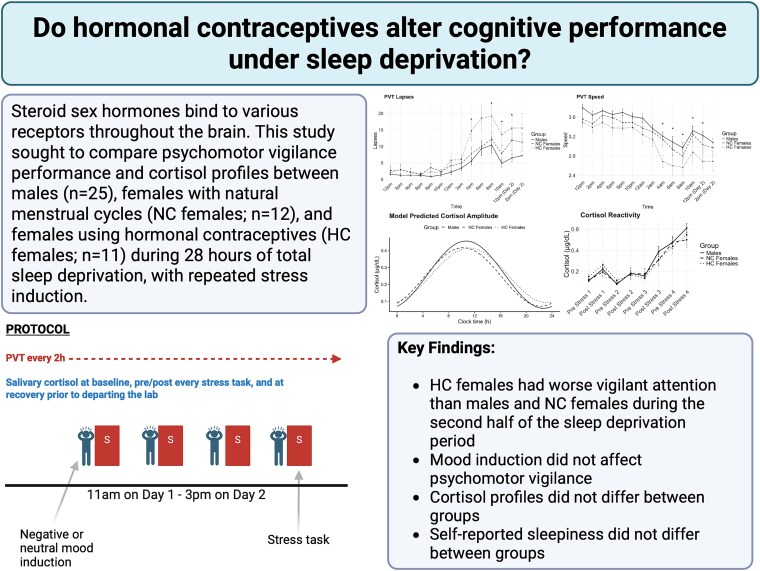

## Introduction

Hormonal contraceptives (HCs) are used by more than 400 million women globally, according to a recent United Nations report [1]. These medications—delivered via pills, intrauterine devices, implants, patches, or injectables—typically function by suppressing endogenous hormone cycling and providing a steady dose of synthetic progestins and/or estrogens. However, the degree of systemic hormone exposure and alteration of endogenous hormone production vary substantially by formulation, with more systemic methods (e.g. oral contraceptives, patches, injectables) producing circulating hormone levels that differ from those associated with intrauterine devices, which primarily exert local effects. While effective for preventing pregnancy, HCs can exert off-target effects on the brain and endocrine system, with consequences for cognition and the stress response [2–4].

Estrogen and progesterone receptors are widely distributed throughout the brain, and both hormones are lipophilic, allowing them to readily cross the blood–brain barrier [5]. In naturally cycling (NC) individuals (those not using hormonal contraceptives), these hormones fluctuate rhythmically across the 24-hour day, and more significantly across the menstrual cycle, inducing dynamic changes in brain structure and function [6, 7]. Neuroimaging studies have documented cycle-dependent changes in gray matter volume, and functional alterations have been observed in salience, default mode, and executive control networks [8, 9]. Fluctuating hormones can also exert effects on the hypothalamic–pituitary–adrenal axis, with cortisol secretion varying across the menstrual cycle [10].

These rhythmic hormonal changes are absent with hormonal contraceptive use, yet the extent to which this alters cognitive performance remains unclear. Relatively few studies have systematically examined their impacts, and findings remain mixed. Some research suggests HC use is associated with adverse mood and cognitive outcomes, particularly among adolescents or those individuals without pre-existing psychiatric symptoms [11, 12], whereas other studies report mental health benefits in populations with premenstrual dysphoric disorder (PMDD) [5, 13]. A recent meta-analysis found that oral contraceptives (OCs) affect multiple cognitive domains beyond simple motor function; however, the direction and magnitude of effects varied depending on the drug formulation, modality, and specific task used [14]. Differences in the level of androgenic, estrogenic, and progestogenic activity likely contribute to these heterogeneous outcomes. Moreover, sensitivity to the action of synthetic progestins on mineralocorticoid and glucocorticoid receptors can alter the secretion of cortisol both across the biological day and in response to stress [4]. Lovallo *et al.* [15] have reported that women using HC exhibited higher resting baseline cortisol levels and blunted cortisol reactivity to stress compared to NC females, while Roche et al. [16] observed a delayed morning cortisol peak among HC users.

Critically, there is a paucity of data on how HCs modulate cognitive performance during sleep deprivation, a condition highly relevant for shift workers, caregivers, and military personnel. Emerging evidence suggests that individuals using HCs may experience worse subjective sleep quality, insomnia symptoms, and sleep efficiency [17, 18]. This might mean that when opportunities to sleep are available, sleep health is impaired among HC women, relative to NC women. However, HCs may improve sleep for individuals with menstrual disorders, such as PMDD, polycystic ovary syndrome (PCOS), or endometriosis, for whom menses may otherwise lead to sleep disruptions due to pain or mood symptoms [19, 20].

In a sample of rotating shift workers, HC users (including a mixture of contraceptive modalities) reported greater fatigue and reduced alertness than both naturally cycling (NC) women and men, but objective psychomotor vigilance task (PVT) performance did not differ significantly between groups [21]. Urrila *et al.* [22] found no PVT performance differences between NC and HC users during sleep deprivation, however HC users reported greater sleepiness after recovery sleep. In a recent study, Grant *et al.* [23] found that when premenopausal females were hypoestrogenized following injection of a gonadotrophin-releasing hormone agonist during their mid-late luteal phase, they had significantly more PVT lapses and greater reaction time than when they were in their mid-late follicular phase, when estrogen was naturally high. Furthermore, they found that experimental sleep fragmentation did not lead to further decrements in PVT performance among individuals when they were hypoestrogenized, while sleep fragmentation did significantly impair these outcomes when estrogen was high. Studies on NC women have identified menstrual phase differences in cognitive performance under sleep deprivation. Contrasting with the findings described above, two studies have shown that women in the follicular phase (when estrogen is high) had more PVT lapses than those in the luteal phase or males during sleep deprivation protocols [24, 25].

Given the endocrine and neurocognitive changes associated with HC use, including altered cortisol secretion and possible suppression of testosterone activity through anti-androgenic formulations, it is important to understand how these factors influence cognitive resilience under physiologic stress. We aimed to address this gap by examining how HC use affects psychomotor vigilance, subjective sleepiness, and cortisol profiles during a controlled 28-hour sleep deprivation protocol. Based on prior research indicating greater fatigue and altered stress physiology among HC users, we hypothesized that (1) PVT performance would be impaired among individuals using HCs compared with NC women and men during sleep deprivation, and (2) individuals using HCs would exhibit reduced cortisol amplitude and a blunted stress response relative to NC women and men.

## Materials and methods

### Participants

A cohort of 48 healthy adults (18-30 years; 23 females) was recruited from the Tucson, AZ region (see Table 1). Participants were recruited via social media, flyers, and newspaper advertisements, in addition to the laboratory website. Eligible participants had no self-reported history of psychiatric or neurological disorder and were fluent in English. Exclusion criteria included pregnancy, history of traumatic brain injury or cardiac conditions, presence of any major medical or chronic medical condition, cognitive impairment, or contraindication to undergoing sleep deprivation, daily nicotine use, or daily caffeine use exceeding 300 mg. Female participants were required to self-report current hormonal contraceptive use or non-use; information on contraceptive formulation (e.g. systemic vs intrauterine) was collected but participants were grouped based on use status. Participants who were not using hormonal contraceptives were asked to provide date of last menses, estimated date of next menses, typical menstrual cycle length, and whether they experienced regular or irregular cycles. Missing data and/or self-reported menstrual cycle irregularity, combined with a lack of biochemical confirmation, precluded accurate menstrual phase estimation for multiple individuals.

**Table 1:** Sample characteristics

		*N*	%
Age	Males (*n* = 25)	22.08 (3.39)	52
	Females (naturally cycling) (*n* = 12)	22.00 (3.72)	25
	Females (hormonal contraceptive users (*n* = 11)	21.36 (3.07)	23
BMI	Males	23.27 (2.60)	
	Females (naturally cycling)	23.28 (2.21)	
	Females (hormonal contraceptive users)	21.44 (3.15)	
Race/Ethnicity	Asian	5	10.5
	Black	4	8.5
	Hispanic/Latino	13	27
	White	22	46
	Unspecified	4	8
Education	High school	9	19
	Some college	23	48
	Bachelor's degree	11	23
	Master's degree	5	10

This study was approved by the University of Arizona Institutional Review Board and the US Army Office of Human Research Oversight (OHRO), and all participants provided written informed consent prior to enrollment.

### Protocol

Data collection for the study occurred throughout 2019. Participants completed study procedures across two laboratory visits separated by 7-10 days. Visit 1 lasted approximately two hours and included a baseline screening and a physical examination from the study physician. Participants provided written informed consent at this visit.

Participants were sent home with an Actiwatch-2 device (Philips Respironics, Murrysville, PA) to collect information about habitual sleep and wake patterns in their home environment for approximately one week prior to returning to the laboratory for the intervention visit (Visit 2). Daily sleep diaries were also completed each morning during this phase.

**Figure 1:**
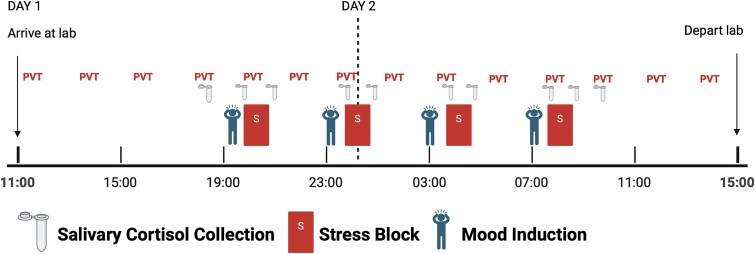
Visit 2: total sleep deprivation timeline.

After at least 1 week of at-home actigraphy and sleep diaries, participants returned to the laboratory for a 28-hour total sleep deprivation (TSD) protocol (Visit 2). A schematic for this visit is detailed in Fig. 1.

Participants were instructed to maintain a fixed sleep schedule for the two nights preceding the in-laboratory TSD visit, with bedtimes between 22:00 and 23:00, and wake times by 08:00, allowing for a total sleep time of 7–9 hours. Compliance was verified upon arrival at the laboratory through inspection of actigraphy records. Subjects were confined to the laboratory for the duration of the study and did not have contact with individuals outside of the laboratory. Subjects did not have access to phones, email, or other forms of electronic communication. Neurobehavioral testing was completed in individual bedrooms, with subjects seated at a desk with a laptop computer. Subjects were allowed to interact with one another and research staff during free time, which was held in a common area. Subjects were monitored continuously by trained research assistants. The laboratory conditions were strictly controlled with light levels fixed at 100 lux and ambient temperatures maintained at an average of 72°F.

During this visit, participants completed 14 standard task batteries administered every 2 hours, between 12:00 on Day 1 through 14:00 on Day 2, via desktop computer. Each battery included a 10-minute Psychomotor Vigilance Task (PVT) preceded and followed by the Karolinska Sleepiness Scales (KSS). The PVT is considered the gold standard measure of cognitive alertness [26]. It presents participants with a millisecond counter at random intervals between 2 and 10 seconds, upon which they must respond by tapping a button as quickly as possible, without making false starts. The KSS is a single-item instrument that prompts participants with a nine-point Likert scale to rate their current level of sleepiness (ranging from 1 = “extremely alert” to 9 = “extremely sleepy”) [27]. Salivary cortisol samples were collected 10 times: at baseline, immediately before and after each stress block, and prior to departure (Figure 1). Participants departed the laboratory at 15:00 on Day 2. At each collection interval, participants were asked to pool saliva in their mouth, before placing a cotton swab under their tongue and allowing the cotton swab to saturate for two minutes. Saturated swabs were transferred to a tube labeled with the participant ID, time, and saliva sample number. Swabs were immediately transferred to a -80°C freezer for storage, prior to cryogenic shipment for analysis. Samples were assayed at the Salimetrics SalivaLab (Carlsbad, CA) using the Salimetrics Salivary Cortisol Assay Kit (Cat. No. 1-3002), with no modifications to the manufacturer’s protocol (intra-assay CV = 4.6 per cent; inter-assay CV = 6.0 per cent; assay range = 0.012-30 ug/dL; sensitivity = 0.007 ug/dL).

Participants completed four stress blocks across the visit, with a mood induction (randomized to either negative or neutral) prior to each. Mood was induced by having participants listen to classical music of the appropriate affect for 15 minutes, followed by reading and reflecting on statements in line with the target mood, and finally written recall of a personal memory that aligned with the mood condition. The stress blocks, administered every four hours starting at 20:00, included the Maastricht Acute Stress Test (MAST). During this task, participants alternated between submerging their non-dominant arm in 0°C water and performing an oral subtraction task in which they counted backwards from 2043 in steps of 17. These tasks were performed at random intervals for random lengths between 30 and 90 seconds, repeated 5 times in each MAST session, lasting for a total duration of approximately 15 minutes. A researcher was present in the room during the MAST and was instructed to maintain a stoic demeanor throughout the session, instructing the participant to restart the oral subtraction task with every error.

### Statistical analyses

All analyses were performed using R (v 4.4.2, R Foundation for Statistical Computing, Vienna, Austria). The primary outcomes were PVT Lapses (responses exceeding 500 milliseconds), Reaction Time (response time in milliseconds), Speed (1000/Reaction Time), and False Starts (premature responses, or those of less than 150 milliseconds). Mean values of each PVT outcome were calculated for each test interval. Secondary outcomes included salivary cortisol (amplitude, which is the difference between the highest and lowest cortisol values within the 24-hour period, and reactivity, which is the difference between cortisol pre vs post each stress block), KSS score, and actigraphic measures of baseline sleep. Descriptive statistics were used to summarize the sample. Mixed effects models (*lmerTest* package) were used to assess group differences (HC females, NC females, and males) in baseline sleep measures. Subsequent models also assessed the effects of group, time, their interaction, and mood induction condition on raw KSS and PVT Speed, log-transformed PVT Lapses, and log-transformed cortisol outcomes. Subsequent models also included cortisol reactivity as a predictor of PVT outcomes. Log-transformations were used to reduce skewness and better meet model assumptions of normality and homoscedasticity. Subject-level variation was included as a random intercept in each of these models. Effect sizes are reported as partial eta squared (ηp^2^) for fixed effects from models, and as estimated mean differences for pairwise comparisons. A cosinor model was used (*GLMMcosinor* package with Gamma distribution) to compare the circadian rhythmicity of salivary cortisol between groups.

## Results

### Sample characteristics

The sample (*n* = 48; 23 females) were healthy young adults (mean age = 21.89 *+* 3.35 years old; mean BMI = 22.84 *+* 2.71 kg/m^2^), 46 per cent of whom identified as White. Eleven of the 23 females were using hormonal contraceptives. Five of these 11 individuals were using combined pills of various formulas, one was using the Implanon device, two were using an intrauterine device, and the modality was not specified for the remaining three persons. Of the naturally-cycling females, three were estimated to be in the follicular phase of their menstrual cycle, three in the luteal phase, and the remaining six were uncharacterized. Detailed characteristics of the sample can be viewed in Table 1. There were no significant differences between groups in baseline demographics.

### Psychomotor vigilance task performance and subjective sleepiness

**Figure 2:**
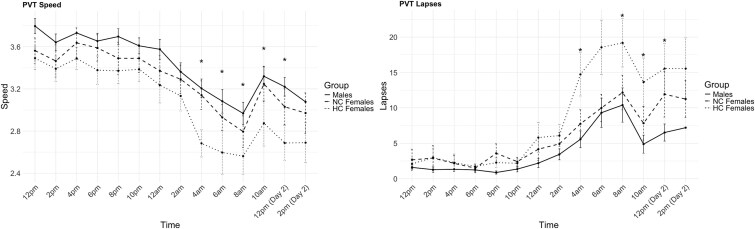
Exploratory group differences by timepoint in PVT lapses and speed. (footnote) *p < .05.

There was a significant effect for group (F(1,45) = 3.75; *p* = .031; ηp^2^ = 0.15), and time (F(13,583) = 47.90; *p* < .001; ηp^2^ = 0.52) but no group-by-time interaction for PVT Lapses (F(26,583) = 1.25; *p* = .183; ηp^2^ = 0.05). Exploratory Bonferroni-corrected pairwise comparisons identified that HC females had significantly more lapses than males at 4 am, 8 am, 10 am, and 12 pm (between 2 and 3-fold greater lapses; all *p* < .030; Fig. 2). A marginal group effect (F(2,44) = 3.05; *p* = .057; ηp^2^ = 0.12), significant time effect (F(13,583) = 53.87; *p* < .001; ηp^2^ = 0.55), and no group by-time-interaction (F(26,383) = 0.90; *p* = .604; ηp^2^ = 0.04) were identified for PVT Speed. Exploratory pairwise comparisons revealed that HC females had significantly longer response times than males at the assessments from 4 am-12 pm (0.41-0.53 units of speed (1000/Reaction Time) slower; all *p* < .050; Figure 2). There was no significant effect of mood on these PVT outcomes. There was no significant difference between groups in PVT Reaction Time (*p* = .469; ηp^2^ = 0.03) nor False Starts (*p* = .568; ηp^2^ = 0.02) or in Karolinska Sleepiness Scale score (*p* = .756; ηp^2^ = 0.01).

### Cortisol secretion profiles

**Figure 3:**
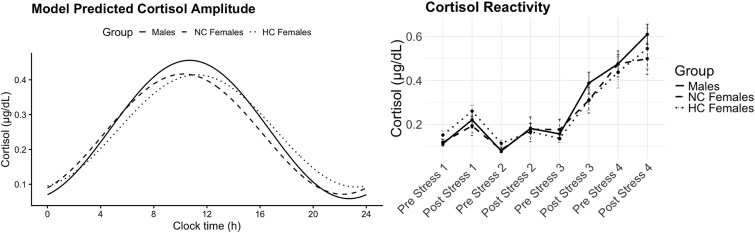
Cortisol secretion profiles. Cosinor analyses performed on cortisol samples collected at baseline (18:40), prior to each stress block (19:30, 23:30, 03:30, 07:30), and recovery (09:00).

Circadian rhythmicity of cortisol profiles was observed in all three groups, with no significant difference between groups. No significant difference in stress reactivity to individual stress blocks, or peak reactivity, was identified between groups (Figure 2) and cortisol reactivity did not predict PVT performance (all *p* > .05). Cosinor comparisons of cortisol profiles between groups suggested a significantly lower cortisol amplitude between males and females using HC (*p* = .046), but this effect did not survive Bonferroni correction for multiple comparisons (p_adj_ > 0.05; Figure 3).

### Actigraphic measures of baseline sleep

Prior to visiting the lab for the TSD portion of the study, participants wore an actigraph for 5-7 days. It was found that NC females slept on average approximately 44 minutes per night than males (F(2,44) = 3.51; *p* = .039; ηp^2^ = 0.15). Total sleep time did not significantly differ between other groups. No significant differences were identified in wake after sleep onset (F(2,39) = 0.48; *p* = .62; ηp^2^ = 0.02).

## Discussion

In this study, females using HC, but not NC females, were found to exhibit poorer PVT speed and a greater number of lapses than males once 20 hours of continuous wakefulness had elapsed until the end of the 28-hour TSD protocol. No significant group differences were observed in self-reported sleepiness (KSS), 24-hour cortisol rhythmicity, amplitude, or stress reactivity, and neither cortisol reactivity nor negative mood induction predicted PVT performance.

Limited research has assessed how HC influences cognitive performance and stress reactivity during TSD. Understanding this is crucial for individuals who perform shiftwork or routinely experience curtailed sleep and circadian disruption, e.g. due to caregiving obligations, emergency response service, or military operations. In contrast to findings by Boivin *et al.* (2023), the present study found no significant group differences in self-reported sleepiness, as measured via the KSS. However, HC females performed significantly worse than other groups in terms of PVT lapses and speed during the latter part of the TSD protocol. This dissociation suggests reduced vigilance efficiency or resilience to sleep loss among HC users that is not fully captured by subjective sleepiness measures [28, 29]. Urrila *et al.* [22] found no significant difference in PVT outcomes during 40 hours of sleep deprivation between 11 NC females in the follicular phase and 22 females using 20 μg ethinylestradiol combined with a third-generation progestin of varying doses. However, those data suggest that there may have been a trend toward worse performance in terms of PVT speed and lapses among HC users, as compared to NC controls, as the TSD period progressed. Urrila *et al.* found that NC females were sleepier than HC-users following recovery sleep, but we were unable to evaluate this in the present study due to not having measured this in participants after recovery.

In the present study, group differences in PVT performance were not observed until the second half of the TSD period, suggesting that HCs may increase vulnerability to acute sleep loss. The mechanisms that may be responsible for this are unclear, but it is possible that this could be due to structural and functional brain changes associated with HC use, alterations to circadian rhythms in cognition, or changes to preceding sleep that resulted in HC users having greater sleep debt prior to entering experimental TSD [14, 17]. A forced desynchrony protocol could be used in future research to measure sleep- versus circadian-driven factors. It is also important to note that HC formulations differentially bind to glucocorticoid and mineralocorticoid receptors. Given that multiple studies have shown that HC can blunt cortisol stress reactivity and decrease overall cortisol amplitude (HC users typically demonstrate higher baseline cortisol during the awakening response), some have speculated that HC place individuals in a state of chronic stress, which may decrease resilience to TSD.

A posthoc power analysis was conducted to determine whether we were powered to detect changes in cortisol reactivity between groups based on previous measures of effect size in this population [30]. It was determined that we were underpowered, which may be why no group differences were identified in this study. Eighteen participants per group would have been required to detect a modest effect size (*d* = 0.7) in line with what has been observed in prior research. Lovallo *et al.* [15] found an interesting time-of-day effect in terms of cortisol and HC use: in the morning, HC users had elevated cortisol levels compared to NC females when at rest, and a dampened response to stress tasks, but this was matched between groups by the afternoon. Cortisol exhibits a diurnal pattern whereby secretion peaks just before the morning awakening and reaches a nadir at night during sleep. HC formulation is likely an important moderator of cortisol activity. Klipping *et al.* [2] found that newer combined oral contraceptive formulas containing drospirenone and estetrol resulted in less of an increase in total cortisol and cortisol binding globulin than more traditional formulas using ethinylestradiol and levonorgestrel. Detailed formulation data were not available for all participants in the present study, limiting further analysis.

Oh *et al.* [31] have shown that among midlife and older women, serum follicular-stimulating hormone (FSH) is positively associated with global cognition, memory, and executive function, independent of circulating estradiol levels. Given that many HC suppress gonadotropins, including FSH and luteinizing hormone (LH), thus preventing ovulation, it is plausible that suppression of endogenous gonadotropins contributes to altered cognitive performance under sleep deprivation as well. Schattmann and Sherwin [32] found that young women with PCOS performed significantly worse than healthy counterparts in various cognitive domains and attributed this to elevated circulating androgens – a hallmark feature of PCOS. HC formulations vary in their binding affinity for androgen receptors [33]. Future research must tease apart the effects of these various formulations on cognition. It is also important to note that certain HCs can dramatically improve quality of life and daytime function for individuals with menstrual disorders, including PCOS, endometriosis, and PMDD [34–36]. There are mixed findings on the effects of hormone replacement therapy on cognitive performance in mid-life women who are navigating menopause, though there are numerous confounds in this population that make it difficult to compare directly to premenopausal individuals [37].

While the directionality of findings is mixed and relatively little research has been conducted in this area, several investigators have observed an association between HC use and impaired sleep [17, 18]. It may be the case that HC use impairs certain domains of cognitive performance and that this effect is mediated by disrupted sleep during available opportunities. However, further research is required to confirm this. It may also be the case that for individuals with menstrual disorders, the attenuation of symptoms often reported during HC use improves sleep. NC females were found to have significantly greater baseline total sleep time than males, but no significant differences were identified between other groups. It is important to note these findings must be interpreted within the context of the small sample size and missing data, which may have precluded an adequately powered comparison between groups. Insufficient information was available on history of menstrual disorders.

This study is one of few to date to investigate how HC use affects PVT performance, self-reported sleepiness, and cortisol amplitude and stress reactivity during TSD. Overall, females taking HC, but not NC females, showed significantly greater vigilance impairment than males during the latter part of overnight sleep deprivation. The small sample size, heterogeneity in HC formulations, and variability in menstrual phase between NC participants prevent conclusive mechanistic associations between HC use and psychomotor vigilance in this study. However, these findings highlight the need for more nuanced investigations of sleep deprivation resilience among individuals using exogenous hormones, with attention to formulation, delivery method, timing of use, and underlying reproductive physiology. The present study was limited by the lack of detailed information on HC formulation and menstrual phase for some of the included individuals. Our hope is that future research will consider these factors and collect information about menstrual phase and HC use to allow for a better understanding of the role of the hormonal milieu in sleep health outcomes and cognitive performance under sleep deprivation.

## Data Availability

Data will be made available upon reasonable request.
